# Elevated vaginal heparan sulfate correlates with impaired neutrophil killing of *Candida albicans* in women with vulvovaginal candidiasis

**DOI:** 10.1128/iai.00709-25

**Published:** 2026-02-09

**Authors:** Junko Yano, Nicole A. Woznicki, Jack D. Sobel, Mary C. Meyaski-Schluter, Paul L. Fidel

**Affiliations:** 1 Department of Oral and Craniofacial Biology, School of Dentistry, LSU Health New Orleans 116781 , New Orleans, Louisiana, USA; 2 Department of Obstetrics and Gynecology, Wayne State University 2954, Detroit, Michigan, USA; 3 Division of Infectious Diseases, Wayne State University 2954, Detroit, Michigan, USA; 4 Clinical and Translational Research Center, LSU Health New Orleans 12258 , New Orleans, Louisiana, USA; University of California Davis, Davis, California, USA

**Keywords:** *Candida albicans*, vulvovaginal candidiasis, inflammation

## Abstract

Recurrent vulvovaginal candidiasis (RVVC), primarily caused by the fungal pathogen *Candida albicans*, is a common infection affecting a significant number of women worldwide. Despite a robust inflammatory response by polymorphonuclear neutrophils (PMNs) with potent antifungal properties during symptomatic episodes, fungal clearance often fails, leading to persistent overgrowth and PMN-associated immunopathology. Studies in an established animal model demonstrated that elevated vaginal heparan sulfate (HS) interferes with PMN-*C. albicans* interactions, thereby impairing fungal clearance. This study investigated the presence and inhibitory effects of HS in women diagnosed with RVVC. Vaginal conditioned medium (VCM) was prepared from swab samples obtained from symptomatic VVC patients, women in asymptomatic remission, and healthy controls. Results from ELISA and immunostaining showed significantly elevated HS levels in VCM-containing vaginal secretions and epithelial cells from symptomatic women compared to those from asymptomatic and healthy controls. PMN killing assays further revealed significantly reduced antifungal activity in the presence of VCM from symptomatic women compared to asymptomatic and healthy control VCM, resulting in a significant negative correlation between vaginal HS concentrations and PMN antifungal activity. The inhibitory effect of HS was further confirmed *in vitro* by impaired PMN killing in control VCM spiked with purified HS, and by the restoration of PMN function following heparanase (HS lyase) treatment of both symptomatic VCM and HS-spiked controls. These findings support the results from the animal studies and provide the first clinical evidence that elevated HS in the vaginal environment contributes to PMN dysfunction, leading to persistent *C. albicans* colonization and VVC-associated immunopathology.

## INTRODUCTION

Vulvovaginal candidiasis (VVC) is a common infection of the vagina primarily caused by the opportunistic fungal pathogen *Candida albicans*. Approximately 75% of women experience at least one episode of VVC during their lifetime, and an additional 5%–8% suffer from recurrent VVC (RVVC) defined as three or more episodes per year (1–3). Known risk factors for VVC include elevated estrogen levels, frequent antibiotic use, and uncontrolled diabetes mellitus (3). However, the majority of RVVC cases are idiopathic, with no identifiable predisposing factors, and often require maintenance antifungal therapy (4–6). Both VVC and RVVC are characterized by hallmark symptoms including itching, erythema, burning, and abnormal discharge, significantly impacting the quality of life for otherwise healthy women worldwide (7, 8).

Extensive investigations into local and systemic adaptive immunity have demonstrated no protective role in VVC (9–14). Instead, growing evidence from clinical studies and animal models highlights the central role of innate immune mechanisms, particularly the involvement of vaginal epithelial cells and polymorphonuclear neutrophils (PMNs) in driving VVC pathology (15–22). Notably, a live challenge study in women demonstrated that the presence of PMNs in the vaginal lumen strongly correlated with symptom severity during acute VVC episodes (23). Subsequent studies in mice have shown that activation of vaginal epithelium by *C. albicans* hyphae, along with damage caused by its cytolytic toxins (e.g., candidalysin), initiates inflammatory signaling, leading to the release of proinflammatory mediators including IL-1β and S100A8 (16, 17, 24, 25). This leads to robust PMN migration into the vaginal lumen that, together with overgrowth of *Candida* hyphae, is ultimately responsible for the characteristic VVC symptoms and associated tissue damage.

Despite their potent antimicrobial properties, migrating PMNs fail to reduce fungal burden in the vaginal environment, but instead amplify inflammation and further contribute to persistent tissue damage. Thus, VVC/RVVC is predominantly the result of an immunopathologic outcome of an otherwise functional PMN response that should be protective. This was exemplified in comparative studies using two distinct mouse strains, VVC-resistant outbred CD-1 mice and VVC-susceptible inbred C3H mice (and several others such as C57BL/6), that revealed distinct outcomes (26). Following inoculation, resistant CD-1 mice efficiently cleared infection through effective PMN antifungal activity including neutrophil extracellular trap (NET) formation (26, 27). This was confirmed by *in vitro* PMN killing assays utilizing vaginal conditioned medium (VCM) consisting of native vaginal secretions of CD-1 mice that exhibited significant PMN-mediated killing (26). In contrast, susceptible C3H mice exhibited persistent colonization and inflammation, concomitant with elevated levels of heparan sulfate (HS) in vaginal secretions, which appeared to mediate PMN dysfunction in the presence of C3H VCM (26). Indeed, a series of *in vitro* mechanistic experiments demonstrated that purified HS inhibited PMN antifungal killing in CD-1 VCM, while heparanase (HPSE, an HS-specific lyase) restored PMN antifungal activity in C3H VCM (26). Collectively, these results indicated that HS acts as a competitive inhibitor for Mac-1 on PMNs, preventing the interaction with Pra1, a pH-regulated antigen on *C. albicans* (28, 29), resulting in impaired PMN-mediated killing. To date, however, this inhibitory action of HS has not been investigated clinically.

HS, a sulfated glycosaminoglycan, is ubiquitously present in various tissues and secretions, including vaginal epithelium and extracellular matrices (30, 31). Human and murine HS has been reported for the ability to bind a wide range of proteins with the potential to act in both inhibitory and activating manners (32–34). Similarly, HPSE is widely expressed locally and systemically, and its enzymatic activity has been shown to regulate HS degradation during tissue remodeling, inflammation, and cell migration (32, 35). Given the previous mouse data, we hypothesized that elevated vaginal HS impairs PMN-mediated fungal clearance during symptomatic VVC/RVVC, which could be reversed by HPSE treatment. Therefore, the objective of this study was to investigate the presence and involvement of HS in impairing PMN antifungal activity in women with symptomatic VVC/RVVC.

## MATERIALS AND METHODS

### Subjects and enrollment

Women diagnosed with VVC/RVVC between the ages of 21 and 55 were recruited over a period between September 2021 and March 2023 at the Infectious Disease Vaginitis Clinic of Wayne State University (WSU). A total of 42 patients who met the age and diagnostic criteria were enrolled at the time of their clinic visits for either acute episodes of VVC/RVVC or maintenance therapy for RVVC. Of those, 30 women had active VVC confirmed by pelvic examination, point-of-care microscopy, positive results from *C. albicans* cultures, and the presence of one or more of VVC-associated symptoms (erythema, edema, burning/rawness/soreness, itching, excoriation, abnormal discharge, odor) at the time of sample collection (symptomatic group). The remaining 12 women were enrolled during an RVVC remission stage, with either positive or negative *C. albicans* cultures, but no symptoms at sampling (asymptomatic group). One subject from each group was excluded from the study due to specimen transport issues. Additionally, five subjects, consisting of three symptomatic and two asymptomatic women, were re-enrolled at follow-up visits where additional samples were collected. Among those, two subjects transitioned from symptomatic to asymptomatic status upon revisits. Cumulatively, a total of 29 symptomatic and 13 asymptomatic subjects were included in subsequent experiments and data analyses.

Healthy women with no history of VVC within the matched age range (n=16) were recruited into the study during the period from August 2021 to April 2023 at the Clinical and Translational Research Clinic of Louisiana State University (LSU) Health-New Orleans.

### Specimen collection and processing

A set of two vaginal swab samples was collected from each subject and pooled. Together with the additional 5 follow-up samples, including the 2 transitional cases, a total of 31 symptomatic and 14 asymptomatic swab samples were obtained. Swab samples from the VVC/RVVC patients at WSU were collected by a physician during pelvic examinations. Control women enrolled at LSU Health-New Orleans performed self-swabbing under clinician guidance. In both cases, vaginal swabbing was conducted using sterile foam-tipped applicators (Puritan) by inserting them into the mid-depth of the vaginal lumen and gently rotating for 10–15 s. Following swabbing, the swab tip was removed from the shaft and immediately placed in a 15 mL conical tube containing 1 mL RPMI 1640 medium (GIBCO) supplemented with 1% penicillin-streptomycin (Invitrogen) and protease inhibitors (Pierce). The procedure was repeated using a second swab. After vortexing to release swab contents into the medium, aliquots were removed and processed for wet mount microscopy for hyphal detection, cultures for quantification of vaginal fungal burden, and species identification as described below. The remaining solution was centrifuged at 1,000 × *g* for 5 min, and the supernatant was sterile-filtered and stored at −80°C as vaginal conditioned medium (VCM). The cell pellet was resuspended in cryopreservation medium (50% fetal bovine serum, 30% RPMI, 20% dimethyl sulfoxide) and stored at −80°C.

### Vaginal fungal burden and *Candida* speciation

To quantify vaginal fungal burden, aliquots from the vaginal swab suspension were serially diluted and cultured on Sabouraud dextrose agar (SDA, BD Diagnostics) supplemented with gentamicin (10 µg/mL, Invitrogen). Colony-forming units (CFUs) were enumerated after incubation for 24 h at 37°C. To determine *Candida* species, additional aliquots were plated onto CHROMagar *Candida* (CHROMagar) and incubated for 48 h at 37°C.

### Microorganism culture

*C. albicans* SC5314 was maintained as a glycerol stock at −80°C. Prior to experiments, a loopful of stock solution was spread onto SDA and incubated at 37°C for 24 h. A single colony was transferred to 10 mL yeast extract-peptone-dextrose broth (BD Diagnostics) and incubated at 30°C with shaking at 200 rpm for 18 h. Stationary-phase *C. albicans* cells were washed three times in sterile phosphate-buffered saline (PBS) by centrifugation at 1,000 × *g* for 5 min and enumerated using a hemocytometer.

### PMN and serum isolation

For PMN isolation, 10 mL of whole blood from healthy volunteers was collected in a sodium heparin vacutainer (BD) tube and mixed with an equal volume of 5% dextran in 0.9% NaCl and incubated for 20 min at room temperature to sediment erythrocytes. Leukocyte-rich plasma was layered over 6 mL Ficoll-Paque Plus (1.077 g/mL density, GE Healthcare) and centrifuged at 400 × *g* for 30 min. The resulting neutrophil-erythrocyte pellet was washed two times in Hanks’ balanced salt solution (HBSS) and resuspended in ice-cold 0.1% NaCl for 30 s to remove remaining erythrocytes by hypotonic lysis. Following washing in HBSS, the neutrophil-enriched pellet was resuspended in 50% human serum in RPMI and maintained on ice until use. PMN viability >95% in the enriched population was confirmed by trypan blue dye exclusion and enumerated using a hemocytometer. For serum isolation, 10 mL of whole blood was allowed to clot for 1 h at room temperature in a serum vacutainer (BD) tube and centrifuged at 1,000 × *g* for 10 min. The serum was aliquoted and stored at −80°C until use.

### VCM

VCM consisted of vaginal swab supernatants filtered through a 0.45-µm pore-size syringe filter. Aliquots were stored at −80°C until use.

### ELISA for HS

Concentrations of HS in VCM samples were measured using a sandwich human HS ELISA (Biomatik) according to the manufacturer’s instructions. Samples were tested in dilutions (1:4–2,000) to fall within the standard curve range. Absorbance was read at 450 nm using a Synergy microplate reader (BioTek).

### Lactate dehydrogenase assay (LDH)

Levels of LDH release in VCM were measured using a CytoTox 96 nonradioactive cytotoxicity assay (Promega) per the manufacturer’s instructions. Samples were tested in dilutions (1:2–10). Absorbance was read at 490 nm on a Synergy microplate reader. LDH activity values are expressed as optical density at 490 nm (OD_490_).

### Immunofluorescence staining

Cell fractions of vaginal swab suspension (1 × 10^4^ epithelial cells/sample) were cytospun onto polysine-coated glass slides (Epredia) using a Cytospin 4 cytocentrifuge (Thermo) at 1,000 rpm for 5 min. After fixation in ice-cold acetone for 5 min, slides were rehydrated in PBS and treated with a blocking buffer (1% bovine serum albumin in PBS) for 15 min, and then incubated with biotinylated anti-HS antibodies (clone F-58 10E4, 5 µg/mL, AMSBIO) overnight at 4°C. Slides were washed, stained with streptavidin-Alexa Fluor 488 (green, Invitrogen) for 30 min and Hoechst 33258 (blue, 1 µg/mL, Thermo) for 15 min, and mounted with Vectashield antifade mounting medium (Vector Laboratories). Cells were examined using an Olympus FV-1000 confocal microscope and FluoView software. To quantify epithelial cell HS expression, the acquired fluorescent images were analyzed using ImageJ software by measuring the cell area, integrated density, and mean gray value of epithelial cells. Corrected total cell fluorescence (CTCF) of each cell was calculated as follows: CTCF = integrated density – (area of selected cell × mean background fluorescent reading) (36, 37). For each subject, 10–14 epithelial cells from three non-adjacent microscopic fields (600×) were evaluated. Data were expressed as mean CTCF per subject.

### PMN-*C. albicans* cocultures and killing assay

PMN antifungal activity was assessed using an *in vitro* PMN-*C. albicans* coculture assay as previously described (26, 38). PMNs were counted and adjusted to 1 × 10^6^/mL in RPMI 1640 medium and transferred to a 96-well plate in a volume of 100 µL per well. The plate was preincubated for 30 min at 37°C with 5% CO_2_ to obtain a monolayer. *C. albicans* blastoconidia were opsonized with 5% human serum for 30 min at room temperature prior to use in cocultures. Following the initial incubation, PMN monolayers (1 × 10^5^/well) were inoculated with *C. albicans* (1 × 10^4^/well) suspended in 100 µL RPMI 1640 medium or VCM samples for 3 h at 37°C with 5% CO_2_. Control wells included *C. albicans* cultured alone in each respective medium. After the coculture incubation, PMNs were lysed with 100 µL of wash buffer (0.05% Triton X in sterile water), followed by washing the well using 100 µL of fresh wash buffer with scraping and aspirating with a pipette tip. The numbers of viable *C. albicans* were assessed by CFU quantification after incubation for 24 h at 35°C on SDA plates. Percent killing was determined in comparison with *C. albicans* cultured alone in each respective medium and calculated as follows: % killing = [1 − (CFUs from PMN-*C. albicans* coculture/CFUs from *C. albicans* alone)] × 100. Results were expressed as % killing ± standard error of the mean (SEM).

### HS spiking and HPSE treatment of VCM

To test inhibitory effects of HS, control (non-inhibitory) VCM was spiked with purified HS (500 µg/mL, from porcine intestine, AMSBIO) prior to coculture. To degrade HS, symptomatic (inhibitory) VCM was pretreated with either recombinant human HPSE (rhHPSE, 4 µg/mL, R&D Systems) or recombinant *Pedobacter heparinus* heparinase III (r*Ph*HPSE, 3 ng/mL, R&D Systems) for 1 h at 37°C. Treated VCM samples were immediately tested in the PMN killing assay. Controls included *C. albicans* cultured alone in HS-spiked or HPSE-treated VCM and used to determine percent killing as described above.

### Statistics

VCM and vaginal epithelial cell samples from each subject were examined individually and included in statistical analyses. For HS-spiking and HPSE digestion assays, VCM samples from two to three subjects were pooled within the groups. Each data set was first tested for normality using the Shapiro-Wilk test. Multiple comparisons were performed using either one-way analysis of variance (ANOVA, parametric) or the Kruskal-Wallis test (non-parametric) followed by appropriate *post hoc* analyses comparing specific groups as indicated in the figure legends. The Pearson product-moment correlation coefficient was used to assess the relationship between HS concentrations and % killing values to determine a linear regression (*R*^2^). Significant differences were defined at a confidence level where *P* < 0.05. All data were plotted and analyzed using Prism software (GraphPad).

## RESULTS

### Demographic characteristics and VVC/RVVC status of study participants

All participants completed a demographic and gynecologic survey prior to sample collection, including age, race, use of antifungal or hormonal therapies, presence of vaginal symptoms, and history of VVC episodes (Table 1). Among the symptomatic women (*n* = 29), all were experiencing active infection with clinical signs and symptoms of VVC confirmed by pelvic examination and laboratory tests. Erythema of the vaginal mucosa was the most frequently reported manifestation of vaginitis in both VVC (*n* = 11, 68.8%) and RVVC (*n* = 11, 84.6%) subgroups, followed by discharge, itching, burning/redness/soreness, and edema. Each symptom occurred at similar frequencies ranging from approximately 43 to 68%. Women in the asymptomatic group (*n* = 13) were enrolled during a remission stage of previously diagnosed VVC/RVVC. A subset of these women (*n* = 5, 38.5%) showed detectable *Candida* colonization despite being symptom-free at the time of sample collection. None of the control subjects without a history of VVC showed detectable *Candida* colonization. Across all groups, use of hormonal contraceptives, HRT, or current pregnancy was rarely reported, with frequencies ranging from approximately 6 to 25%.

**TABLE 1:** Demographics of study participants

		Control (*n* = 16)	VVC/RVVC^*a*^ asymptomatic (*n* = 13)	Symptomatic (*n* = 29)	
				VVC (16)	RVVC (13)
Age	18–25	18.8%	7.7%	12.5%	15.4%
	26–40	43.8%	53.8%	62.5%	69.2%
	41–55	37.5%	38.5%	25.0%	15.4%
Race	Caucasian	87.5%	84.6%	37.5%	46.2%
	African American	6.3%	15.4%	62.5%	53.8%
	Hispanic or Latino	6.3%	0	0	0
	Other	0	0	0	0
Antifungal prophylaxis	Fluconazole	N/A^*i*^	84.6%	12.5%	7.7%
	Boric acid		0	6.3%	7.7%
	None		15.4%	81.3%	84.6%
Hormonal status	Contraceptives^*c*^	12.5%	7.7%	25.0%	7.7%
	IUD^*d*^	6.3%	15.4%	0%	7.7%
	HRT^*e*^	6.3%	7.7%	6.3%	0
	Pregnant	0	0	6.3%	0
	Unmedicated/natural cycles^*f*^	75.0%	69.2%	62.5%	84.6%
VVC^*b*^ symptoms	Asymptomatic	N/A	92.3% (10)	0	0
	Erythema		0	68.8% (11)	84.6% (11)
	Discharge		0	68.8% (11)	53.8% (7)
	Itching		0	50.0% (8)	61.5% (8)
	Burning/redness/soreness		0	56.3% (9)	46.2% (6)
	Edema		0	43.8% (7)	53.8% (7)
	Excoriation		0	12.5% (2)	0
	Odor		7.7% (1)	0	0
Number of episodes in the past 12 months	>3	N/A	23.1%	0	100%
	≤3		23.1%	81.3%	0
	0		53.8%	18.7%	0
Laboratory tests	*C. albicans*	N/A	38.5%	100%	100%
	NAC^*g*^		0	0	0
	Hyphae		0	75.0%	92.3%
	Concurrent conditions^*h*^		7.7%	0	15.4%

^*a*^ RVVC, women diagnosed previously with RVVC defined as four or more acute episodes in a 12 month period.

^*b*^ Values in parentheses indicate the number of subjects who selected each applicable symptom.

^*c*^ Including oral and injectable methods.

^*d*^ Intrauterine device.

^*e*^ Hormone replacement therapy.

^*f*^ Including post-hysterectomy.

^*g*^ Non-albicans *Candida* species.

^*h*^ Including mixed infections with VVC and bacterial vaginosis or seborrheic dermatitis.

^*i*^ N/A, not applicable.

### Relative vaginal pathology in symptomatic VVC and asymptomatic remission

**Fig 1:**
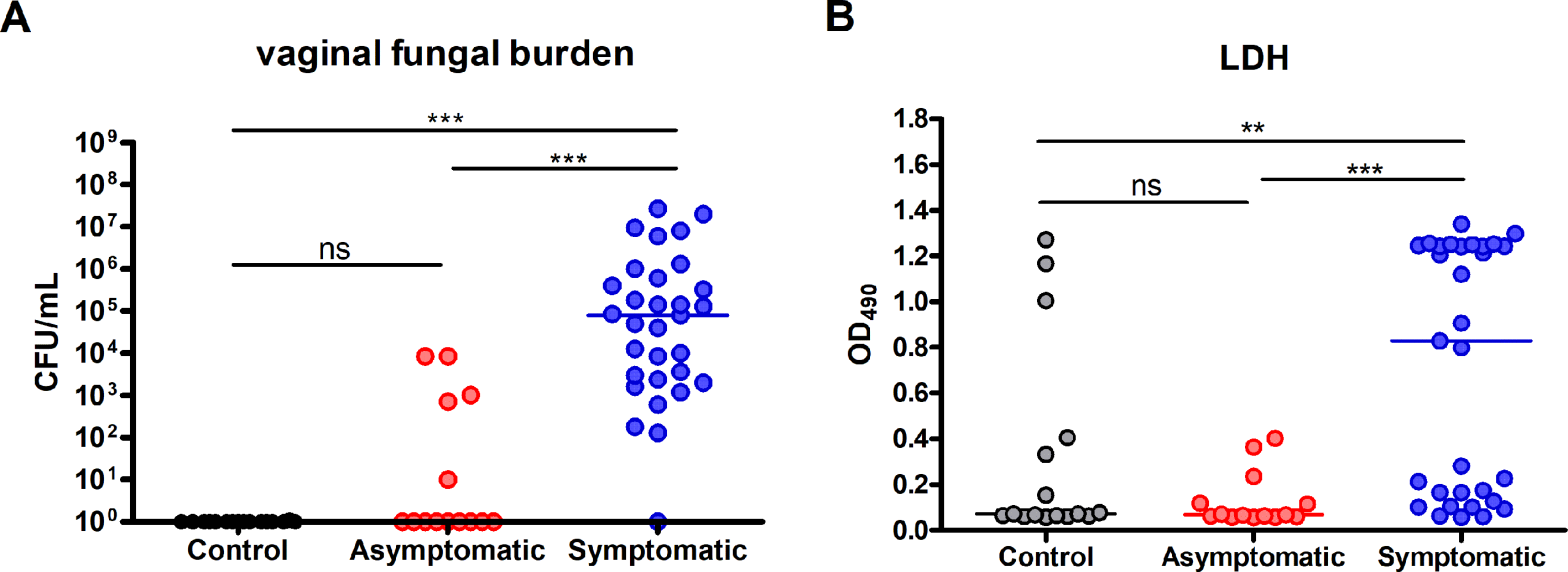
Vaginal fungal burden and tissue damage during acute symptomatic VVC and asymptomatic remission. Vaginal foam-tipped swab samples were collected from three groups: RVVC women experiencing a symptomatic acute episode of VVC (symptomatic group, *n* = 31), those in asymptomatic remission (asymptomatic group, *n* = 14), or healthy women without a history of frequent VVC (control group, *n* = 16). Sample sets included five follow-up swabs collected from women in the symptomatic and asymptomatic groups (three sets within the same group and two spanning both groups). Detached swab tips were suspended individually in 1 mL RPMI 1640 medium to release vaginal secretions and cellular content. Supernatants were then sterile-filtered as VCM. (**A**) Vaginal fungal burden was quantified by culturing pre-filtration swab suspensions on agar and counting CFUs. (**B**) Levels of LDH in VCM were measured as a marker of epithelial damage. Horizontal bars indicate median values for each group. Data were analyzed using the Kruskal-Wallis test and Dunn’s post-test. **, *P* < 0.01; ***, *P* < 0.001; ns, not significant.

To validate the association between clinical signs and symptoms, *C. albicans* load, and vaginal tissue damage, a comparative evaluation of fungal burden and LDH release (as a marker of tissue damage) was initially conducted. As expected, all samples from the symptomatic group, except one obtained during a follow-up visit, contained significantly elevated fungal burden compared to those from the asymptomatic and control groups (Fig. 1A). In contrast, only a small subset of the asymptomatic group exhibited low to moderate levels of fungal colonization. All isolates were identified as *C. albicans*. Notably, *C. albicans* was predominantly found in the hyphal form during microscopy in most symptomatic cases (*n* = 24, 77.4%), whereas hyphae were absent in all asymptomatic samples, despite positive cultures in some instances. Similarly, LDH levels in the supernatants were significantly elevated in the symptomatic group compared to the asymptomatic and control groups (Fig. 1B). Additionally, a small number of control samples showed LDH levels comparable to those of symptomatic samples despite no detectable fungal colonization.

### HS is significantly elevated during symptomatic VVC/RVVC episodes

**Fig 2:**
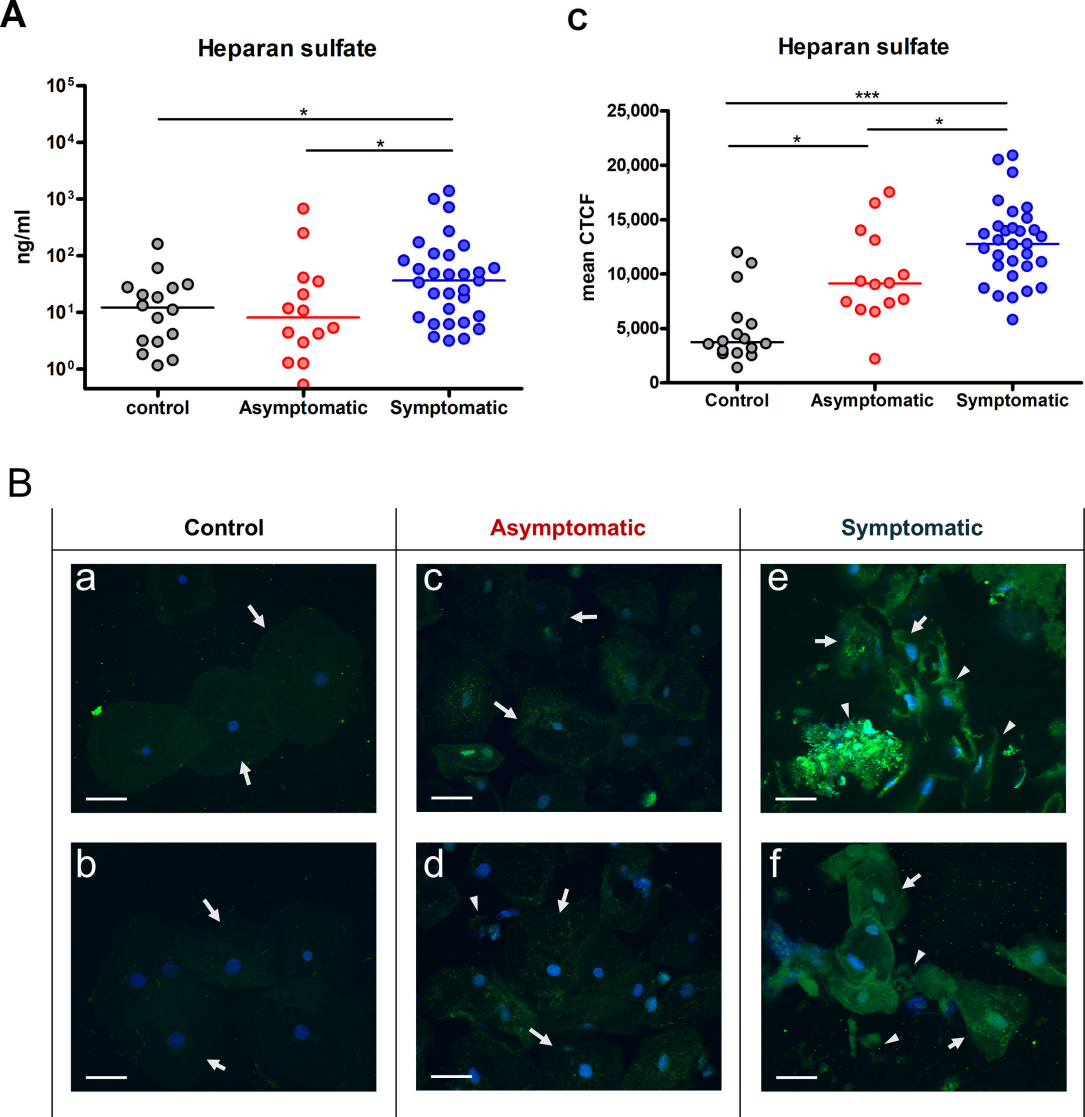
Quantification and epithelial cell expression of HS in vaginal specimens from women with VVC. Vaginal swab samples were collected from three groups: women with RVVC experiencing a symptomatic acute episode of VVC (symptomatic group, *n* = 31), those in asymptomatic remission (asymptomatic group, *n* = 14), and healthy women without a history of frequent VVC (control group, *n* = 16). The data sets included an additional five follow-up swabs collected from women in the symptomatic and asymptomatic groups (three sets within the same group and two spanning both groups). Detached swab tips were suspended individually in 1 mL RPMI 1640 medium to release vaginal secretions and cellular content. Supernatants were sterile-filtered as VCM. (**A**) Levels of HS in VCM were measured by quantitative ELISA. (**B**) Cytospin preparations of epithelial cells (1 × 10^4^/sample) from pre-filtration swab suspensions were stained with biotinylated anti-HS antibodies (clone F-58-10E4, 5 µg/mL) followed by streptavidin-Alexa Fluor 488 (green). Cell nuclei were counterstained with Hoechst 33258 dye (1 µg/mL blue) and observed by confocal microscopy (600× magnification, scale bars measure 50 µm). Arrows indicate epithelial cells. Arrowheads denote epithelial debris when present. Two representative images from each group are shown: a and b, control; c and d, asymptomatic; e and f, symptomatic. (**C**) HS expression in epithelial cells (green fluorescence) was quantified by measuring CTCF in 10–14 epithelial cells from three non-adjacent microscopic fields per sample. Each dot represents the mean CTCF value per sample. Horizontal bars in dot plots indicate group medians. Data were analyzed using the Kruskal-Wallis test and Dunn’s post-test. *, *P* < 0.05; ***, *P* < 0.001.

Previous studies in a mouse model showed that vaginal HS can act as a competitive analog of the PMN Mac-1 receptor, thereby inhibiting the interaction of Mac-1 with its ligand on *C. albicans*, Pra1, and impairing PMN-mediated fungal recognition and killing (26). Based on this, it was hypothesized that elevated HS contributes similarly to impaired fungal clearance in women. To test this clinically, HS levels were assessed in vaginal swab samples using two complementary approaches. First, measurements of secreted HS in VCM by ELISA revealed significantly increased HS concentrations in the symptomatic group (Mdn = 36.7 [8.2–102.3] ng/mL) compared to the asymptomatic (Mdn = 8.1 [2.1–38.3] ng/mL and control (Mdn = 12.2 [3.1–27.5] ng/mL) groups (Fig. 2A). Secondly, immunofluorescence staining showed minimal HS expression in epithelial cells from the control group (Fig. 2B, a and b), moderate and localized staining in the asymptomatic group (Fig. 2B, c and d), and markedly elevated HS expression in the symptomatic group, shown by intense cytoplasmic staining (Fig. 2B, e and f). Cells from the symptomatic group also displayed morphological abnormalities, characterized by disrupted cell membranes and loss of the smooth, intact cell surface typical of healthy sloughed epithelial cells in controls. To validate the staining results, quantification of green fluorescence intensity in epithelial cells confirmed significantly higher HS expression in both symptomatic and asymptomatic groups compared to controls, and with significantly higher levels in the symptomatic group compared to the asymptomatic group (Fig. 2C).

### PMN antifungal activity is inhibited under symptomatic VVC conditions and correlates with elevated HS

**Fig 3:**
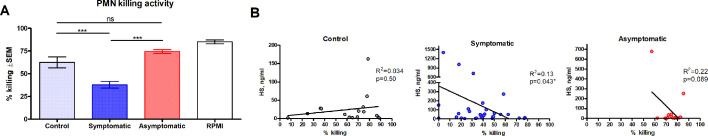
PMN antifungal activity in VCM from women with symptomatic VVC, asymptomatic remission, or no history of VVC. Vaginal swab samples were collected from three groups: women with RVVC experiencing a symptomatic acute episode of VVC (symptomatic group, *n* = 31), those in asymptomatic remission (asymptomatic group, *n* = 14), and healthy women without a history of frequent VVC (control group, *n* = 16). The data sets included an additional five follow-up swabs collected from women in the symptomatic and asymptomatic groups. Detached swab tips were suspended individually in 1 mL RPMI 1640 medium to release vaginal secretions and cellular content. Supernatants were sterile-filtered as VCM. PMNs (5 × 10^5^) isolated from peripheral blood of healthy volunteers were examined for *in vitro* killing activity against *C. albicans* by culturing with 1 × 10^5^ yeast cells in 100 µL of VCM or RPMI medium for 3 h at 37°C with 5% CO_2_. *C. albicans* cultured alone in VCM or RPMI medium were used to calculate % killing. Viable *C. albicans* cells were enumerated by quantitative plate counts. (**A**) PMN killing activity in VCM derived from symptomatic, asymptomatic, or control groups. (**B**) Correlation between HS concentrations in VCM and PMN antifungal activity in control (left panel), symptomatic (center panel), or asymptomatic (right panel) groups. Regression lines are shown for each scatter plot. Data were analyzed using the one-way ANOVA and Bonferroni’s post-test (**A**) or the regression correlation analysis (**B**). Bar heights and error bars (**A**) represent the group means ± SEM for % killing values across independent replicates of unique VCM samples. *, *P* < 0.05; ***, *P* < 0.001; ns, not significant.

To evaluate the functional capacity of PMNs in the vaginal environment, a PMN killing assay was conducted using subject-derived VCM. Results revealed that PMNs cultured in asymptomatic and control VCM maintained strong killing capacities similar to those in standard RPMI medium alone (74.4 ± 2.07% and 62.5 ± 6.01%, respectively). In contrast, PMNs exposed to symptomatic VCM showed significantly reduced antifungal activity compared to the asymptomatic and control groups, reaching only 37.8 ± 3.64% killing (Fig. 3A). Given the observed elevated HS levels in symptomatic VCM, a regression analysis was conducted to evaluate the relationship between HS concentrations and PMN activity in each group. Results indicated a significant negative correlation in the symptomatic group, whereas no appreciable correlation was found in the asymptomatic or control groups (Fig. 3B). Additionally, a separate regression analysis was performed comparing epithelial cell HS expression with PMN antifungal activity. Although the symptomatic group showed a moderately negative correlation similar to that obtained with levels of secreted HS concentrations in VCM measured by ELISA, the trend observed in CTCF values did not reach statistical significance (data not shown).

### HS inhibits PMN antifungal activity in vaginal environment

**Fig 4:**
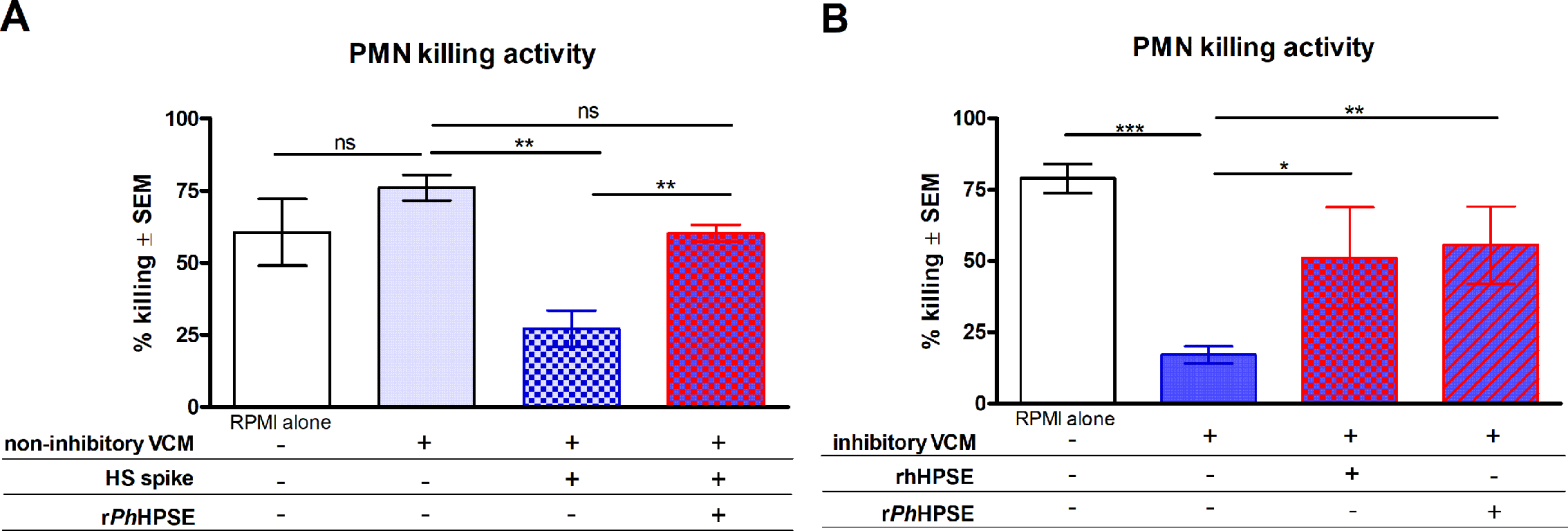
PMN antifungal activity in VCM spiked with HS or treated with HPSEs. Vaginal swab samples were collected from three groups: women with RVVC experiencing a symptomatic acute episode of VVC (symptomatic group, *n* = 31), those in asymptomatic remission (asymptomatic group, *n* = 14), and healthy women without a history of frequent VVC (control group, *n* = 16). The data sets included an additional five follow-up swabs collected from women in the symptomatic and asymptomatic groups. Detached swab tips were suspended individually in 1 mL RPMI 1640 medium to release vaginal secretions and cellular content. Supernatants were sterile-filtered as VCM. PMNs (5 × 10^5^) isolated from peripheral blood of healthy volunteers were incubated with *C. albicans* cells (1 × 10^5^) in 100 µL of VCM or RPMI medium for 3 h at 37°C with 5% CO_2_. Controls consisted of *C. albicans* cultured alone in VCM or RPMI medium and were used to calculate % killing. (**A**) VCM samples with normal killing activity (non-inhibitory VCM, gray bar) were spiked with purified porcine HS (500 µg/mL) and reevaluated for PMN killing activity (blue bar). Spiked non-inhibitory VCM was then pretreated with r*Ph*HPSE (3 ng/mL) and reevaluated for PMN killing activity (red bar). (**B**) VCM samples with low killing activity (inhibitory VCM, solid blue bar) were pretreated with rhHPSE (4 µg/mL, red-dotted bar) or r*Ph*HPSE (3 ng/mL, red-striped bar) and reevaluated for PMN killing activity. After coculture, viable *C. albicans* cells were enumerated by quantitative plate counts. Data were analyzed using the one-way ANOVA and Bonferroni’s post-test. Bar heights and error bars represent group means ± SEM for % killing values across independent replicates of unique VCM samples. The graphs represent cumulative data from three to four experiments. *, *P* < 0.05; **, *P* < 0.01; ***, *P* < 0.001; ns, not significant.

Given the observation that elevated HS levels were associated with reduced PMN antifungal activity under symptomatic VVC conditions, it was important to determine the inhibitory role of HS within the vaginal environment. To this end, several mechanistic experiments were conducted to directly evaluate the impact of HS on PMN antifungal activity. First, VCM samples previously confirmed for supporting normal PMN killing capacity (non-inhibitory VCM) were spiked with purified HS and evaluated in the standard PMN killing assay. Results showed that the addition of purified HS to non-inhibitory VCM led to a significant reduction in PMN antifungal activity from a pre-spiking baseline of 76.1 ± 4.48% to 27.2 ± 6.34% (Fig. 4A). To determine whether this inhibitory effect was reversible, HS-spiked VCM samples were subsequently treated with recombinant HPSE (rHPSE), an HS-degrading lyase originally identified in *Pedobacter heparinus*. Results indeed showed that HPSE treatment of HS-spiked VCM restored PMN fungal killing activity to levels comparable to the pre-spiking baseline (60.23 ± 2.92%) (Fig. 4A). Building on the observed ability of HPSE to reverse HS-induced inhibition of PMN activity, a similar approach was used to evaluate whether HPSE treatment could restore PMN function in naturally inhibitory VCM from symptomatic women. Similar to the observation with HS-spiked non-inhibitory VCM, pretreatment of inhibitory VCM with r*Ph*HPSE or rhHPSE led to significantly enhanced PMN antifungal activity, increasing from a pretreatment baseline of 17.1 ± 3.02% to 55.6 ± 13.6% (r*Ph*HPSE) and 51.1 ± 17.8% (rhHPSE) (Fig. 4B). To validate the extent of enzymatic HS degradation in VCM, an enzyme activity assay was conducted using symptomatic VCM samples as a substrate, and HS concentrations pre- and post-HPSE treatment were quantified by ELISA. Results indicated a >70% reduction in HS levels following treatment with r*Ph*HPSE, reducing from 103.9 ± 36.6 ng/mL pre-treatment to 19.7 ± 3.8 ng/mL post-treatment.

## DISCUSSION

Despite the wide availability of antifungal agents for clinical use, including treatment for VVC and RVVC, standard therapies often provide only transient symptom relief and frequently fail to prevent recurrences or resolve symptoms in cases of resistance (6, 39). This gap underscores the critical need to understand the complex interplay between host immune factors and *C. albicans* that drive symptomatic infection beyond fungal burden alone. In the current study, we focused on the role of HS, a host-derived ubiquitous glycosaminoglycan, as a putative inhibitory factor of PMN antifungal activity, in the human vaginal environment. Our previous work in a mouse model indicated that vaginal HS blocked PMN-*C. albicans* interactions, specifically the binding of Mac-1 on PMNs to Pra1 on *C. albicans*, thereby inhibiting the ability of the infiltrating PMNs to kill *Candida* despite robust recruitment (26). A series of mechanistic experiments further showed that the addition of exogenous HS impaired standard PMN-mediated fungal killing, whereas HS degradation by HPSE restored PMN function.

Building on this foundation, the current study provides the first clinical validation of a similar mechanism in human samples. Using patient-derived VCM, results revealed a functional association between elevated vaginal HS levels and impaired PMN-mediated fungal killing during symptomatic infection. Importantly, enzymatic degradation of HS by recombinant HPSEs restored PMN function under both natural and HS-spiked inhibitory conditions, providing direct evidence for a causal role of HS in mediating PMN dysfunction and immunopathogenesis in human VVC. This is also the first study to report both secreted and cell-associated expression of HS in human vaginal mucosa.

While HS involvement in positive physiological roles has been documented, including wound healing, tissue repair, and epithelial integrity (40–42), excessive HS has also been implicated in chronic inflammation and cancers, where it blocks leukocyte adhesion, disrupts chemokine gradients, and suppresses immune cell signaling (35, 43–45). To date, a role for HS had not been implicated in mucosal fungal infections outside of our work in vaginal candidiasis. In the vaginal mucosa, it is plausible that constitutive levels of HS may contribute to homeostatic protection by limiting mild tissue damage related to menstruation or intercourse. However, an interesting hypothesis is that under stress conditions of *Candida* overgrowth/hyphal formation, HS overproduction or dysregulation adversely influences PMN effector functions, thereby reducing antifungal activity and promoting symptomatic vaginitis.

The paradoxical hyperinflammatory yet ineffective PMN antifungal activity observed during VVC may reflect that HS-mediated interference with Mac-1-Pra1 binding redirects PMN effector functions away from *Candida*. Consequently, PMN degranulation, oxidative burst, and the release of antimicrobial products are likely directed toward the vaginal mucosa rather than the fungal targets via NETs (27). This misdirected response may preserve, or even amplify, proinflammatory properties that drive the immunopathogenic response. Future mechanistic studies will examine broader PMN activity at varying HS levels to further elucidate how changes in the vaginal environment contribute to the dysfunctional, symptomatic state.

Clinically, this dysfunctional/misdirected response may play a role in those women who remain symptomatic despite antifungal therapy, particularly among those prone to recurrence or poor response to azole treatment (6, 46). Importantly, the ability of HPSE to restore PMN function under inhibitory conditions *in vitro* suggests some therapeutic potential for HS-targeting approaches. Because concentrations of endogenous vaginal HS in symptomatic women measured by ELISA were substantially lower than those of the purified porcine-derived HS required in mechanistic assays, HS degradation by HPSE may offer greater effectiveness within this lower physiological range and sufficiently restore PMN function in the vaginal cavity. In the current study, VCM samples from symptomatic women contained HS concentrations of approximately 100 ng/mL, which appeared sufficient to limit PMN antifungal activity. Importantly, HPSE treatment reduced these HS levels by nearly 72%, achieving significant restoration of PMN antifungal activity *in vitro*.

Our findings from the asymptomatic group also offer insights into physiological vaginal immune tolerance to *C. albicans* in colonized women. *C. albicans* was detectable in the absence of hyphae, inflammation, or elevated HS and PMNs in their respective VCM exhibited relatively normal function *in vitro*. These results highlight the concept that colonization alone does not trigger HS secretion or an immunopathogenic response unless *Candida* undergoes hyphal transition with candidalysin production and associated inflammatory signaling (17, 47, 48). These findings align with previous reports that hyphal growth and candidalysin production, rather than fungal presence alone, are key determinants of VVC immunopathogenesis (48).

Unexpectedly, results indicated substantial inter-subject variability in HS and LDH levels across all groups. Although both HS and LDH were elevated in the symptomatic group, no strong correlation was found between the two markers, suggesting that HS elevation is not solely induced by epithelial damage. This may reflect other contributing factors, such as epithelial turnover, hormonal influence, host genetics, and vaginal microbiota composition, none of which were formally evaluated in this cohort. Attempts to correlate HS levels with menstrual phase were also inconclusive (data not shown). Future studies should incorporate the evaluation of hormonal status (i.e., menstrual cycles, hormonal contraception, pregnancy) to formally analyze vaginal HS levels under defined conditions, as these factors likely have a strong influence on broader vaginal mucosal immunity (49–51). Furthermore, while HS levels showed a significant negative correlation with PMN killing, the correlation coefficient was modest, suggesting that additional factors, such as host proteases or microbial products within the vaginal microenvironment, may also influence PMN function.

While the current findings are intriguing and clinically relevant, several limitations in the study design should be noted. First, real-time PMN quantification in VCM samples was not feasible due to the sampling protocol that prioritized fluid recovery and preservation. While smear microscopy provided qualitative confirmation of PMN presence, consistent enumeration was unreliable due to dilution in the transport medium. Additionally, the cross-sectional design restricted sampling to a single time-point analysis, limiting the ability to assess the kinetics of HS release, PMN function, and symptomatology over the course of infection. Although follow-up samples were available in select cases, longitudinal sampling could provide a more comprehensive evaluation and help elucidate these relationships. Finally, although the current study focused on VVC and RVVC, the role of HS in other vaginal conditions, such as bacterial vaginosis (BV) or BV-VVC mixed infections, remains unexplored (52–54). Given that HS has been implicated in bacterial and viral infections of the female genital tract via microbial adherence and interference with immune cell function (33, 34, 55), it is plausible that vaginal HS may act as a common mediator or a marker of inflammatory dysregulation. Whether HS overproduction represents a homeostatic protective response that becomes non-beneficial, or a pathological outcome from chronic inflammation or recurrent infection, remains unknown, although we speculate that HS levels are elevated prior to detectable inflammation. As such, it is conceivable that *C. albicans* may actively promote HS release or inhibit HPSE activity as part of an immune evasion mechanism as the inflammatory response is triggered. Indeed, *C. albicans* has been shown to interact with various host glycosaminoglycans to facilitate adhesion and biofilm formation (56, 57).

The current findings raise important diagnostic and therapeutic potential. Local HS accumulation may serve as a non-culture-based biomarker to distinguish active infection from asymptomatic colonization, particularly for determining inflammatory and non-inflammatory conditions (22, 23). As demonstrated, elevated HS could predict symptomatic outcome with poor PMN function or delayed/reduced clearance, offering a new parameter for therapeutic guidance. In such instances, adjunct therapies targeting local HS, such as topical HPSE treatment or a HS-neutralizing agent, possibly in combination with antifungal therapy, may help restore PMN function, augment host-mediated fungal clearance, and ultimately promote faster transition back to homeostasis. Therapeutically, localized delivery of HPSE may have an advantage over conventional antifungal agents by minimizing any disruption in commensal flora and/or reducing resistance (58–60). However, the safety and effectiveness of such interventions *in vivo* remain to be investigated, including long-term effects of HS degradation on vaginal homeostasis.

In conclusion, this study demonstrates the first clinical evidence that elevated vaginal HS is associated with impaired PMN antifungal activity in women with symptomatic VVC/RVVC, supporting prior data in a mouse model. This immune inhibitory effect is functionally reversible via enzymatic degradation, establishing HS as a newly defined predisposing factor for infection. As such, HS represents both a potential biomarker and therapeutic target in VVC/RVVC. Future research should aim to develop practical screening methods for vaginal HS detection, investigate HS regulation in the vaginal mucosa, the presence of HS during mixed infections, and ultimately to explore HPSE-based therapeutic delivery strategies that would offer more personalized and consistent care for affected women.
